# Automated Generation of Supported Lipid Bilayer Arrays with Controlled Receptor Densities in Well Plates

**DOI:** 10.1021/acsami.6c02551

**Published:** 2026-03-30

**Authors:** Jannis Schlicke, Jacopo Movilli, Dorothee Wasserberg, Raphael N. Biendara, Samer Aphrham, Pascal Jonkheijm, Elif Uslu, Geert-Jan P. H. Boons, Robert Molenaar, Rick Elbert, Sjaak de Wit, Erhard van der Vries, Jurriaan Huskens

**Affiliations:** † Department of Molecules and Materials, MESA+ Institute and TechMed Centre, Faculty of Science and Technology, 3230 University of Twente, P.O. Box 217, Enschede 7500 AE, The Netherlands; ‡ Department of Chemical Biology and Drug Discovery, Utrecht Institute for Pharmaceutical Sciences, 8125 Utrecht University, 3584 CG Utrecht, The Netherlands; § NanoBioPhysics Group, MESA+ Institute, University of Twente, 7500 AE Enschede, The Netherlands; ∥ Royal GD, Arnsbergstraat 7, Deventer 7418 EZ, The Netherlands; ⊥ Complex Carbohydrate Research Center, University of Georgia, 315 Riverbend Road, Athens, Georgia 30602, United States; # Department of Chemistry, University of Georgia, Athens, Georgia 30602, United States

**Keywords:** supported lipid bilayers, receptor density control, fluorescence quantification, multivalent interactions, glycan arrays, DNA hybridization, biosensing

## Abstract

Understanding the superselective, multivalent interactions that drive immunity, infection, and biosensing requires (i) model surfaces with precisely tunable receptor density and (ii) quantitative readouts. Here, we introduce a fully automated well-plate-based platform using cell-mimicking supported lipid bilayers (SLBs) that enables both requirements with standardized, commercially available equipment. We outline the main challenges associated with integrating liquid handling with the shear and disruption-sensitive SLB system and offer practical guidelines to overcome them. The resulting versatile and scalable workflow yielded high-quality antifouling surfaces without laborious manual pipetting, while preserving the simplicity of pipet and well-plate-based liquid handling. It enables complex assays requiring more than 1000 aspiration and dispensing steps over >12 h while maintaining surface integrity. Control over the receptor density was achieved by variation of the molar fraction of a biotin-functionalized lipid inside the SLB, to which streptavidin and biotinylated receptors were bound using the strong biotin–streptavidin interaction. A quantitative readout of the receptor density employing fluorescently labeled streptavidin was statistically validated in the low pmol·cm^–2^ range. The generated arrays were applied in two biorecognition studies requiring elaborate liquid-handling procedures. DNA hybridization showcases a strong, specific, and stoichiometric binding of fluorescently labeled complementary DNA. Furthermore, the platform was established as an alternative type of glycan array capable of studying the complex, multivalent binding of labeled virus particles scalable to the high-throughput processing of samples.

## Graphical abstract

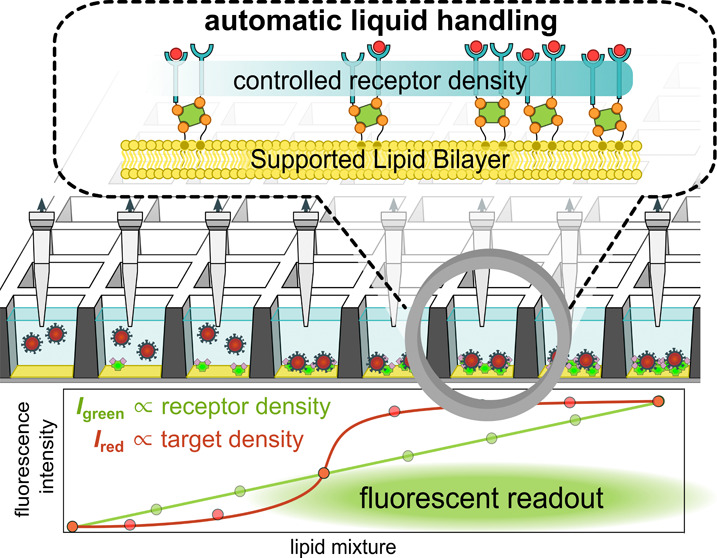

## Introduction

Many biological processes rely on multivalent interactions, where multiple weak and cooperative binding events allow for the high selectivity required for and observed in binding events that result in biological interactions and functions.^1,2^ The exquisite selectivity observed in weakly interacting biological systems can be attributed to superselectivity,^3,4^ i.e., the strongly nonlinear dependence of binding on receptor density. Such binding events are characterized by a sharp sigmoidal dependence on receptor density, with substantial binding occurring only above a critical threshold receptor density.

Superselective binding processes are a key part of the immune system^1,5^ as well as cell–bacteria interactions^6–8^ and viral attachment.^9–11^ Gaining insight into these complex, multivalent processes is crucial for both elucidating pathogenic pathways, virus–host interactions and zoonotic potential^10–12^ as well as for the design of highly site-specifically targeting drugs.^2,13^ Furthermore, multivalency is integral to many biosensing approaches, ranging from antibody^14^ and virus assays^9,10,12,15,16^ to nanoparticle-based sensors,^17–19^ as it enables the high sensitivity and selectivity essential for early disease detection.^20–22^

The study of superselective binding processes requires model systems with precisely tunable receptor densities and a quantitative readout of both ligand and receptor density. Although groundbreaking advances have been made in recent years,^9,12,19,23–28^ they often depend on complex syntheses or specialized methodologies. This paper presents a straightforward, scalable approach for quantitative receptor density control based on supported lipid bilayers (SLBs).

Due to their antifouling and cell-mimicking properties, SLBs are a well-established platform for studying biorecognition processes.^12,15,28–30^ However, their scalable and reproducible fabrication has been undermined by poor air stability and shear sensitivity as well as frequently inconsistent formation.^31,32^ Many existing SLB assays—especially those involving arrays of SLBs—rely either on patterned surfaces, or on specialized assay platforms based on microfluidics. Early studies used lithographically patterned substrates to achieve partitioned SLB corrals,^33^ which would later be individually addressed.^34,35^ Subsequently, microfluidic gradient generators have been used to establish lateral gradients in membrane composition or ligand surface density based on the precise control of flow conditions or electrophoresis.^12,36–42^ More recent work has shown that highly multiplexed microarrays,^36^ fabricated through microfluidic routing, can give valuable insights into complex research questions such as cell signaling. Despite these promising advances, these assays rely on specialized chip fabrication, setups and workflows which are difficult to scale or adapt to each individual research question. Moreover, the standardization of microfluidics is complex and in its early stages.

In reality, much preliminary work and general research still relies on the SLB preparation based on laborious manual pipetting, which implicates a lack of consistency.^43–45^ To overcome susceptibility to human error, while maintaining flexibility in the choice of materials and ease of implementation, only a limited number of approaches have been made to fabricate SLBs by automated, robotic processing. Robotic array printers were successfully applied to generate arrays of lipid spots, which could later be rehydrated to form SLBs, which is only applicable to certain substrates or lipids.^46,47^ Kaufmann et al.,^48^ on the other hand, presented a more broadly applicable method based on noncontact printing through a thin surface confined water film, which involves specific sample handling.

To overcome the limitations outlined above, this study introduces the first scalable protocol for the fully automated generation of SLBs in well plates using standardized laboratory equipment and automated liquid handling (see Table S1). In line with the growing trend toward robotic laboratories,^44,45,49–52^ our approach minimizes operator bias and enhances reproducibility, thereby enabling standardized and scalable SLB preparation. The protocol is accompanied by a user guide (detailed methodology and process parameters) and troubleshooting strategies. Furthermore, we present an effective and straightforward method for the systematic variation of receptor densities, which is quantitatively assessed and statistically evaluated. Finally, we demonstrate the broad applicability of the method by applying it to two representative cases of biorecognition: DNA hybridization and virus binding to glycan arrays.

## Results and Discussion

### Fully Automatic Generation of SLBs by Automated Liquid Handling Protocol

Here, we present a fully automated workflow for the preparation and functionalization of SLBs in well plates, which is illustrated in Figure 1. We aim to highlight its benefits for applications in scientific questions involving complex biorecognition processes. First, we demonstrate the protocol for the SLB formation by vesicle fusion using an automatic liquid handler, and discuss defects and optimizations required to obtain homogeneous, defect-free bilayers from DOPC/biotin-DOPE mixtures. Second, we show how the biotinylated lipid fraction (*x*_bio_) enables precise control over the receptor density through streptavidin (SAv) binding, as established in earlier studies.^28^ The range and precision of this variation is quantitatively and statistically assessed through the fluorescence measurements of dye-labeled streptavidin (SAv-AF488 and SAv-AF350) using both microscopy and plate reader readouts. Third, we apply the established platform to DNA hybridization, using biotinylated DNA probes as receptors and fluorescently labeled complementary and noncomplementary strands as targets. Finally, we extend the approach to a more complex research scenario: the binding of fluorescently labeled PR8 influenza A virus particles to biotinylated glycan receptors. Owing to the excellent control of the receptor density, the superselective nature of this multivalent binding can be quantified, demonstrating the applicability of the method to recognition processes on cell-mimicking surfaces.

**Figure 1:**
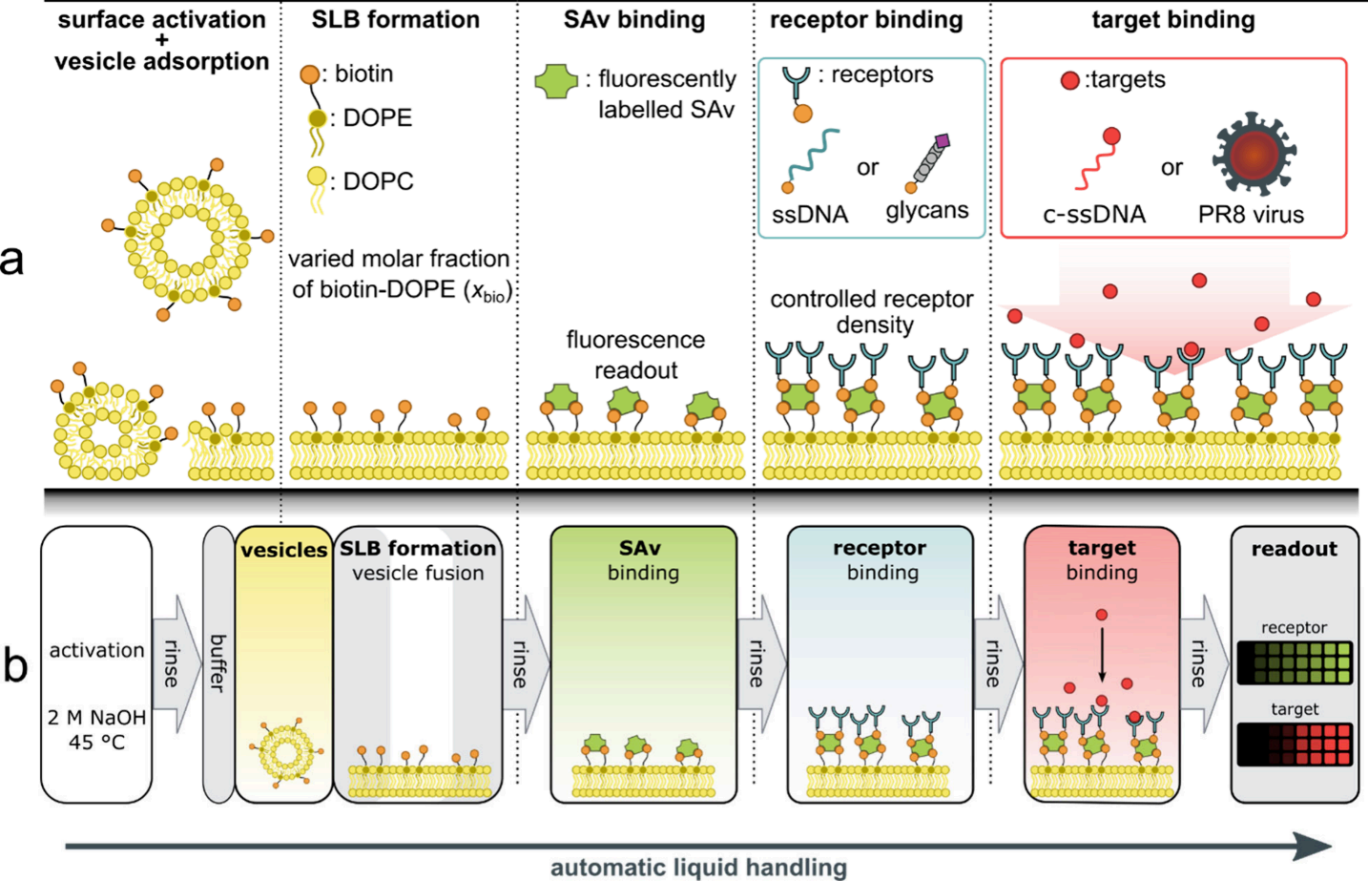
Concept of the generation of arrays of SLBs with controlled receptor densities by automatic liquid handling in well plates. The functional surfaces can be employed in biorecognition assays to study, for example, multivalent binding processes. (a) Schematic representation of SLBs prepared by the fusion of vesicles containing a mixture of the lipids DOPC and biotin-DOPE. The molar fraction of biotin-DOPE is varied to tune the surface density of subsequently bound SAv and biotinylated receptors. Molecular structures and further information about the receptors and targets can be found in Figure S1. (b) Process flow diagram describing the individual steps involved in the process. Major steps of the process are separated by thorough rinsing protocols to remove excess reagents.

Automated SLB formation was implemented on a liquid handler equipped with an 8-tip head and a temperature-controlled stage accommodating 96- and 384-well flat glass-bottom plates. The general workflow (Figure 1b) was designed to mimic common manual pipetting protocols and involved six main steps: (1) surface activation and vesicle adsorption, (2) SLB formation through vesicle fusion, (3) binding of fluorescently labeled streptavidin, (4) receptor binding, (5) target binding, and (6) readout. In this section, we focus on the first two steps, covering the automatic generation of SLBs, which required systematic optimization. During process development, several recurring defects were observed and subsequently resolved. Representative examples of these problems, their causes and the corresponding solutions will be discussed based on Figure 2a–d.

**Figure 2:**
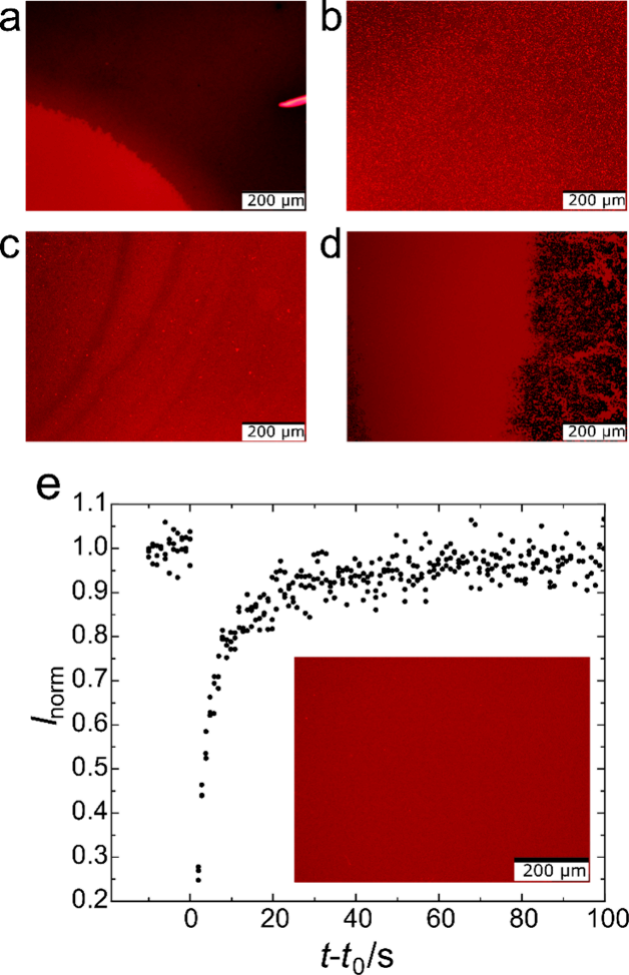
Fluorescence micrographs showing common defects in SLBs (99.7% DOPC, 0.3% TR-DHPE) encountered during well-plate array process development, which exemplify results of unsuitable parameters for the liquid handling: (a) negative edge defect resulting from insufficient wetting during activation; (b) positive defects showing unruptured vesicles; (c) defects due to excessive shear force; (d) delaminated SLBs due to air exposure. (e) FRAP data obtained for a high-quality, homogeneous SLB formed by the optimized automatic pipetting protocol (99.7% DOPC, 0.3% TR-DHPE) showing the fluorescence recovery after the bleaching event at *t*_0_. The inset shows the corresponding micrograph obtained at 10× magnification.

The first problem was incomplete SLB coverage, where only the center of the well was coated while the edges remained unfunctionalized (Figure 2a). This was most problematic for the square wells of 384-well plates and was attributed to incomplete wetting of the glass surface during NaOH activation, caused by insufficient solution volume or suboptimal activation conditions. Complete surface coverage was achieved by using 30 μL of 2 M NaOH at 45 °C for 60 min for 384-well plates.

The second problem was incomplete vesicle rupture during the 30 min incubation with 100 nm lipid vesicles (0.13 g·mL^–1^), leaving residual unruptured liposomes of higher local intensity on the surface (positive defects) and resulting in inhomogeneous bilayers (Figure 2b). These defects were attributed to insufficient driving force for vesicle rupture, which contrasts findings for QCM-D experiments using flow-based functionalization.^15,59^ The issue was resolved by introducing extensive rinsing steps, including salinity switches between PBS buffer (137 mM NaCl, pH 7.3) and ultrapure water. Consistent with earlier reports,^52^ the osmotic pressure shocks promoted complete rupture and minimized positive defects.

The third problem was bilayer damage characterized by circular, concentric defects around the position of the pipet tip (Figure 2c). This was attributed to excessive shear forces during pipetting, linked to both the vertical positioning of the tip and an excessive dispensing rate. Stable bilayers were obtained by lowering the pipetting speed from 100 to 5 μL·s^–1^ and carefully adjusting the *z* position to 1.5 mm above the well bottom to allow for efficient rinsing without disturbing the SLB. Automatic liquid-level detection was found unsuitable, since the critical factor is the distance between the tip of the pipet and the bottom of the well, whereas liquid levels vary with evaporation, particularly in longer protocols.

The fourth and most severe problem was delamination of SLBs leading to lower intensity patches (negative defects), which was primarily observed around the pipet tip positions in the well centers (Figure 2d). This defect was attributed to the introduction of air bubbles into the system, as SLBs are highly unstable to air exposure. To prevent this, particular care was taken to ensure a fully air bubble-free system, during both instrument preparation and liquid handling. It is especially crucial to avoid blowout or air-gap volumes, since this will directly affect the surface quality.

The stability of the SLBs during the workflows was determined by the handling conditions rather than by the absolute duration of the experiments. In particular, exposure to air, excessive shear forces during pipetting or manual handling and repeated temperature fluctuations were found to be most crucial. Throughout this study, all SLBs were kept continuously hydrated and sufficient buffer was supplied to avoid the drying out of the membranes. In addition, well plates were covered and stored in a temperature controlled, dark space to reduce evaporation and photobleaching of fluorescent labels. These measures proved effective and allowed a stable SLB surface even during extended protocols with up to more than 1000 aspiration/dispensing steps in each well over the course of more than 12 h. Throughout this study, a total of 600 SLBs in 25 individual experiments in 384-well plates were prepared. 97% of the surface preparations proved suitable for analysis. A preparation was regarded suitable for analysis, when the majority of the surface was coated with a homogeneous SLB, as shown in Figure 2e, allowing for a quantitative analysis. When stored in a dark, temperature-controlled environment, the SLBs were found stable for multiple days to more than a week.

With these optimizations in place, the workflow reproducibly yielded homogeneous SLBs in a fully automated fashion. The detailed protocol is provided in the Supporting Information. Briefly, liquid handling was performed with precisely calibrated equipment to maintain a constant distance between the pipet tips and the well bottom. Pipetting speeds were kept low and any introduction of air into the wells or tips was prevented. After activating the glass surfaces in NaOH at 45 °C, lipid vesicle dispersions were added and the SLB formation by vesicle rupture was promoted by buffer changes resulting in osmotic stress. Subsequently, the stepwise functionalization with SAv, biotinylated receptors and complementary targets was carried out in PBS with sufficient incubation and rinsing steps, using low pipetting speeds and careful positioning of the tips to minimize damage to the SLBs.

The quality of the layers was furthermore confirmed by fluorescence recovery after photobleaching (FRAP) measurements (Figure 2e). The observed half-recovery times (τ_1/2_) of 2.27 ± 0.19 s have previously been observed by us under comparable conditions for homogeneous, low-defect DOPC SLBs and indicate good layer quality.^15^ For general comparability, diffusion coefficients were determined following the method by Kang et al.,^53^ which takes into account that diffusion is already taking place during bleaching. This correction is certainly necessary as the average half-recovery time (2.27 ± 0.19 s) is of the same order of magnitude as the bleach time (1.6 s) and significant diffusion has taken place between bleaching and recording the first image after bleaching. The average diffusion coefficient determined using the above method amounted to 2.67 ± 0.20 μm^2^·s^–1^, which is in excellent agreement with earlier reports of diffusion constants of supported DOPC bilayers in the range of 1–4 μm^2^·s^–1^.^54–58^ Overall, these results confirm the development of a robust, highly reproducible method to fabricate SLB arrays of excellent quality.

### Well-Plate Arrays of SLBs with Controlled Receptor Densities

With the SLB array method established, we functionalized the cell-mimicking surface using the chemistry described in Figure 1 and utilized it to control the surface density of receptors. The molar ratio of the biotinylated lipid in the vesicles (*x*_bio_) was varied between 0% and 2%. As reported previously,^28^ this range is sufficient to achieve up to full streptavidin coverage. The resulting SAv-coated SLBs are used subsequently to bind biotinylated receptors. The resulting SAv (θ_SAv_) and receptor densities (θ_rec_) can therefore be effectively tuned by the density of the biotinylated lipid in the SLB (θ_bio_), which can be derived from the lipid surface density θ_lipid_ based on the molecular footprint of a DOPC lipid according to previous studies^15,28^ using eq 2 (see Methods). SLBs were incubated for 45 min with 50 nM fluorescent SAv conjugate (AF488-SAv or AF350-SAv). The incubation time is fully sufficient for the SAv–biotin binding as can be estimated from QCM-D experiments (Figure S2) and the literature.^15,59^ After the removal of the excess volume, thorough rinsing was required to avoid the interference of the fluorescence originating from labeled SAv remaining in the supernatant. Bilayer integrity and homogeneity were preserved after the functionalization and rinsing steps (Figure 3a).

**Figure 3:**
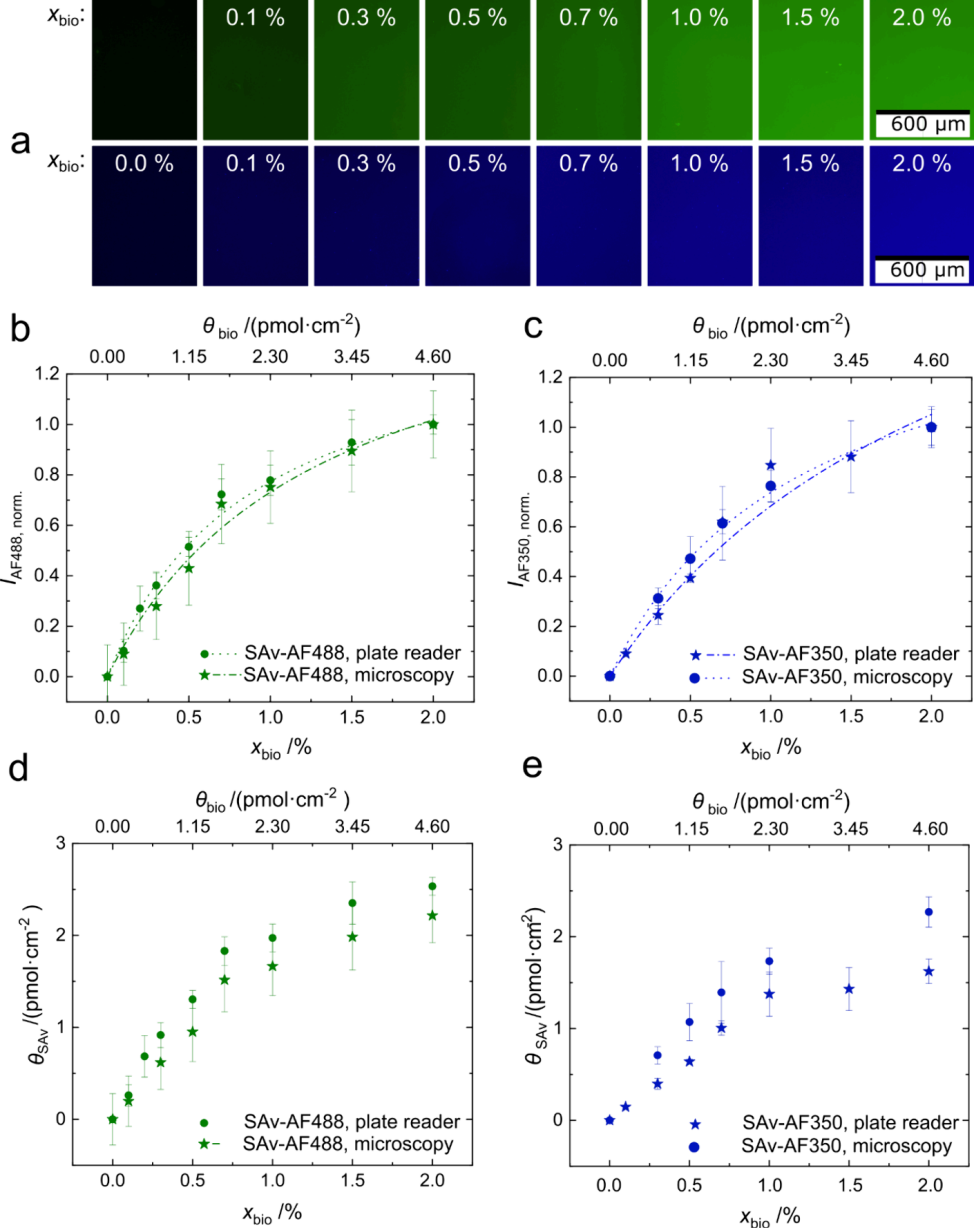
Control of biotin receptor density on DOPC/biotin-DOPE SLBs with different *x*_bio_ after the binding of (green) SAv-AF488 or (blue) SAv-AF350. (a) Fluorescence micrographs visualizing the density of bound SAv. (b and c) Quantitative analysis of the fluorescence intensities obtained for SAv-AF488 and SAv-AF350, respectively, by microscopy and plate reader. Normalized average pixel intensities (min–max scaling to intensities at *x*_bio_ = 2% and *x*_bio_ = 0%) are shown as a function of *x*_bio_ (bottom *x* axis) and θ_bio_ (top *x* axis), which was obtained according to eq 2. A minimum of three independent experiments (each triplicated) was performed for each data set. Errors represent the propagated standard deviations from individual measurements and averaged data sets. Langmuir fits are included as dotted lines (eq 3). (d and e) Molar surface density of SAv (θ_SAv_) as a function of *x*_bio_ (bottom *x* axis) and θ_bio_ (top *x* axis). θ_bio_ was derived from panels b and c by an intensity weighed proportionality relative to a fully saturated surface.

By using fluorescently labeled streptavidin, the SAv density could also be assessed by fluorescence microscopy and plate reader measurements. The fluorescence micrographs shown in Figure 3a exhibit the increasing SAv density as a function of *x*_bio_ for the two different fluorescent conjugates SAv-AF488 (a, top) and SAv-AF350 (a, bottom).

A more quantitative analysis of the SAv densities is shown in Figure 3b–e. Fluorescence intensities increased with *x*_bio_ and approached saturation at approximately *x*_bio_ = 1.5%, which is in agreement with previous studies.^28^ The maximum normalized fluorescence intensities corresponding to a saturated layer, were extrapolated by fitting the binding curves (*I*_AF488,norm._ and *I*_AF350,norm._ as a function of *x*_bio_ in Figure 3b,c; fits are shown as broken lines) with Langmuir isotherms. Molar surface densities of SAv (Figure 3d,e) were estimated from relative, normalized fluorescence intensities (*I*_norm._ *I*_norm.,max_^–1^) as a fraction of the maximum SAv density (3.7 pmol·cm^–2^).^28^ Details concerning this quantification can be found in the Methods section (eqs 3 and 4).

Similarly increasing fluorescence intensities as well as SAv densities as a function of *x*_bio_ are observed for SAv-AF488 and SAv-AF350 (Figure 3). Readout by fluorescence microscopy and plate reader gave similar results with some variation at lower coverages (*x*_bio_ < 1%). Both methods are feasible for a quantitative analysis, while only microscopy gives a verification of the good quality of the SLBs as possible defects can be directly seen. The good repeatability of the described method enables a consistent quality, which justifies the much faster and more easily automatable readout by plate reader.

The choice of fluorophore proved to be much more important than the readout method. AF488 gave smaller deviations as compared to AF350. Based on the data presented in Figure S3, the limits of detection (LOD) were determined from a linear approximation of data for low *x*_bio_ (Figure S3 and Table S2). Thus, determined LOD values for θ_bio_ are 0.023 ± 0.007 and 0.20 ± 0.01 pmol·cm^–2^ for AF488 and AF350, respectively. For low coverages, the corresponding SAv densities (θ_SAv_) can be approximated as 0.5θ_bio_, as is evident from the fluorescence intensities.

The differences in LOD depending on the fluorophore highlight the benefit of using fluorophores with higher quantum yields, as discussed above. However, both LOD values are within the range relevant for applications in biorecognition.^12,23,28^ For studies requiring precise control of receptor densities at the low density range, AF488 is recommended. In general, it can be assumed that fluorophores with a high quantum yield and emission wavelength and low background fluorescence from substrates and materials are superior. Practically, however, the choice of fluorophore is often limited by the availability of labeled targets as well as the setup.

In conclusion, these results demonstrate that the receptor density on SLBs can be precisely tuned by adjusting the biotinylated lipid fraction and reliably quantified by fluorescence readout. The fully automated method provides reproducible, high-quality, cell-mimicking, antifouling surfaces with a sensitivity down to the low pmol·cm^–2^ range, providing a robust basis for subsequent biorecognition studies.

### DNA Hybridization

We demonstrate the use of the density-controlled SLB platform shown above for two separate biorecognition studies. As a first showcase representing high-affinity, highly selective binding, we quantify DNA hybridization on SLBs prepared by a fully automated workflow. SLBs were functionalized with SAv-AF350 and incubated with different biotinylated DNA oligomers as probes (Figure 1a). Biotinylated DNA oligomers of both a sequence complementary (c-ssDNA) to the target ssDNA as well as a scrambled sequence (non-c-ssDNA, open symbols) were used as probes to prove the specificity of the subsequent target binding. Next, the hybridization of the AF488-labeled target DNA sequences to the DNA probes was investigated as a function of probe density (see the schematics in Figure 4a and b).

**Figure 4:**
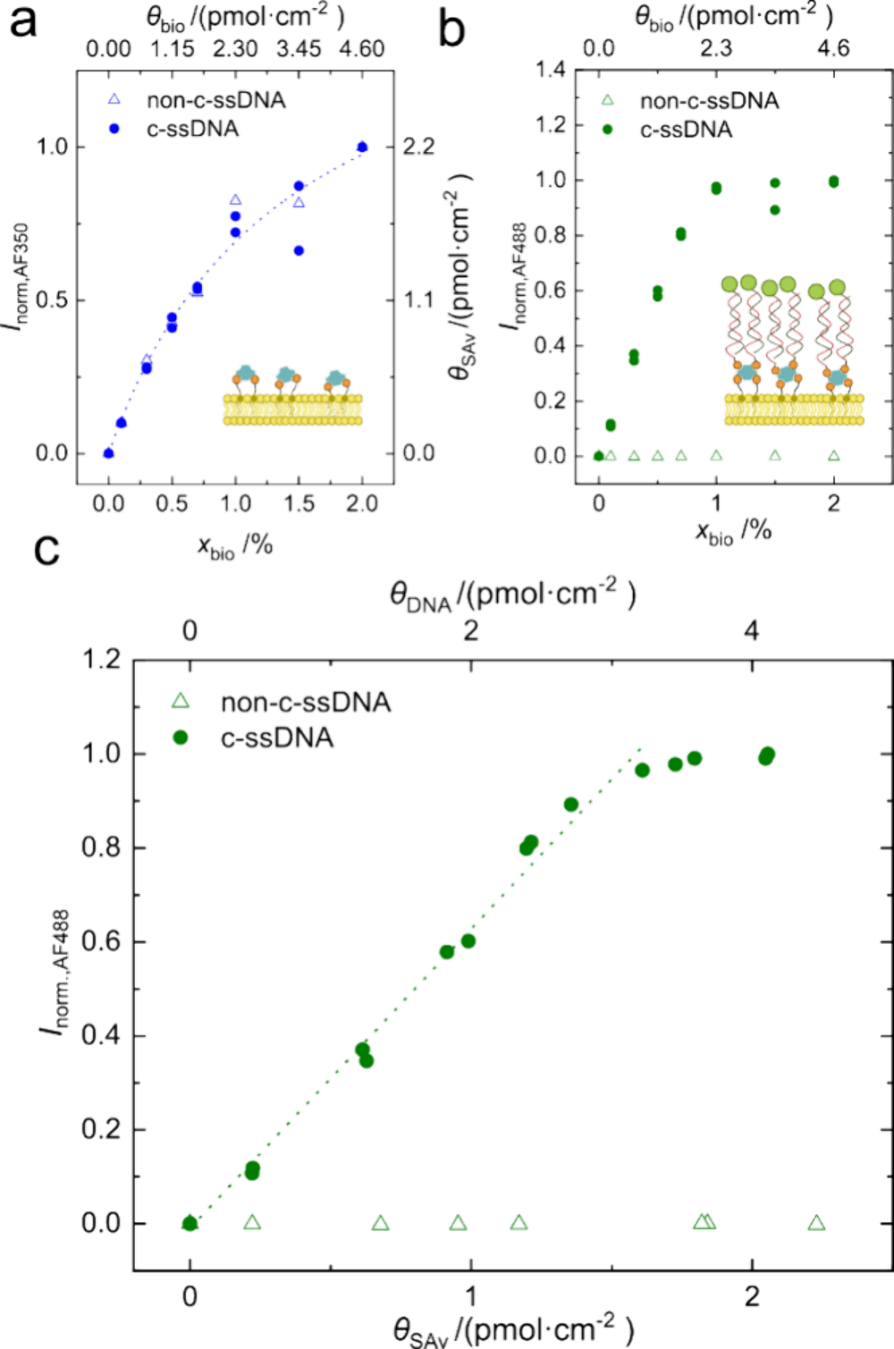
DNA binding and hybridization as a function of probe density. The biotinylated probe DNA (c-ssDNA) was bound to SAv-AF350, which was adsorbed to DOPC/biotin-DOPE SLBs with varying *x*_bio_. A biotinylated, scrambled, noncomplementary probe DNA (non-c-ssDNA, open symbols) is included as a negative control. Such DNA-functionalized surfaces were then incubated with the target DNA fluorescently labeled with AF488 (AF488-ssDNA). The normalized fluorescence intensities (pixel averages) measured for the AF350-labeled SAv and the AF488-labeled target DNA after the completion of the assay are plotted as a function of *x*_bio_ in panels a and b, respectively. SAv densities (θ_SAv_) derived from the normalized fluorescence intensities of the SAv-AF350 according to the previously described procedure (Figure 3) are represented as a second ordinate in panel a. The corresponding fit used for the extrapolation of the saturation of the SAv binding is shown as a dotted line in panel a. The target binding as a function of both SAv density (θ_SAv_) as well as the probe density (θ_DNA_) is illustrated in panel c for both the complementary (c-ssDNA) as well as the noncomplementary DNA (non-c-ssDNA). The probe density is assumed to equal θ_DNA_ = 2θ_SAv_.

Using the process described above, the variation of probe density with *x*_bio_ was achieved, as witnessed by the increasing fluorescence intensity from SAv-AF350 (Figure 4a). Similarly, the fluorescence intensity from the fluorescently labeled complementary DNA increases with *x*_bio_, while no such increase is observed for fluorescently labeled noncomplementary DNA (Figure 4b). This shows the selectivity of the hybridization as well as the good antifouling properties of the DOPC SLBs toward DNA.

The fluorescence intensity attributed to the target DNA as a function of the SAv density, to which the probe DNA was bound, yields further quantitative and density-dependent information about the hybridization. For this purpose, the SAv densities were determined from the SAv intensities according to the previously described procedure (Figure 3a). The resulting plot of the target DNA density quantified by the fluorescence intensity from the AF488 fluorophore as a function of θ_SAv_ and the probe density θ_DNA_ is shown in Figure 4c. The DNA probe density is assumed to equal 2θ_SAv_.

A near-linear regime (dotted line in Figure 4c) for the target binding as a function of the probe density followed by a plateau at high probe densities (θ_SAv_ > 1.6 pmol·cm^–2^) can be seen. This indicates that optimal DNA hybridization is limited to a probe DNA density below the maximum investigated density. This can be attributed to electrostatic repulsion, which is in agreement with the literature.^60^ The corresponding maximum probe DNA density that allows full hybridization can thus be estimated as θ_DNA_ = 2θ_SAv_ = 3.2 pmol·cm^–2^.

Altogether, this density-resolved method provides a general tool to map and tune the operating window of these hybridization assays by identifying the probe density range that preserves linear, specific target binding.

### Well-Plate-Based Assay for Virus Binding

Next, we applied the array methodology to multivalent virus-glycan binding. The binding of virus particles to glycosylated surfaces is an active field of research and poses a set of challenges to surface chemistry. As compared to the highly specific binding of DNA, quantitative virus binding studies require better antifouling properties due to the more prevalent nonspecific binding of protein-dense particles. As will be discussed later, this is especially relevant for “field samples” that can also contain aggregates of virus particles and residual proteins from the media they are obtained from. SLBs formed from zwitterionic lipids like DOPC provide the required low background fouling. In this section we will describe the receptor density-dependent binding of PR8 influenza A virus particles to glycan-functionalized SLB surfaces.

Following the scheme in Figure 1a, SLBs with tunable SAv density were functionalized with different biotinylated glycans. We examined PR8 binding to two glycan receptors: (LN)_2_ (negative control) and 2,6-S­(LN)_3_ (α-2,6-sialylated; expected PR8 binder). All incubation and rinsing steps were fully automated. Exemplary micrographs of these virus binding studies are shown in Figure S4. The fluorescence intensities from the SAv as well as the labeled virus particles, determined by fluorescence microscopy are shown in panels a and b of Figure 5, respectively. The results are summarized in Figure 5.

**Figure 5:**
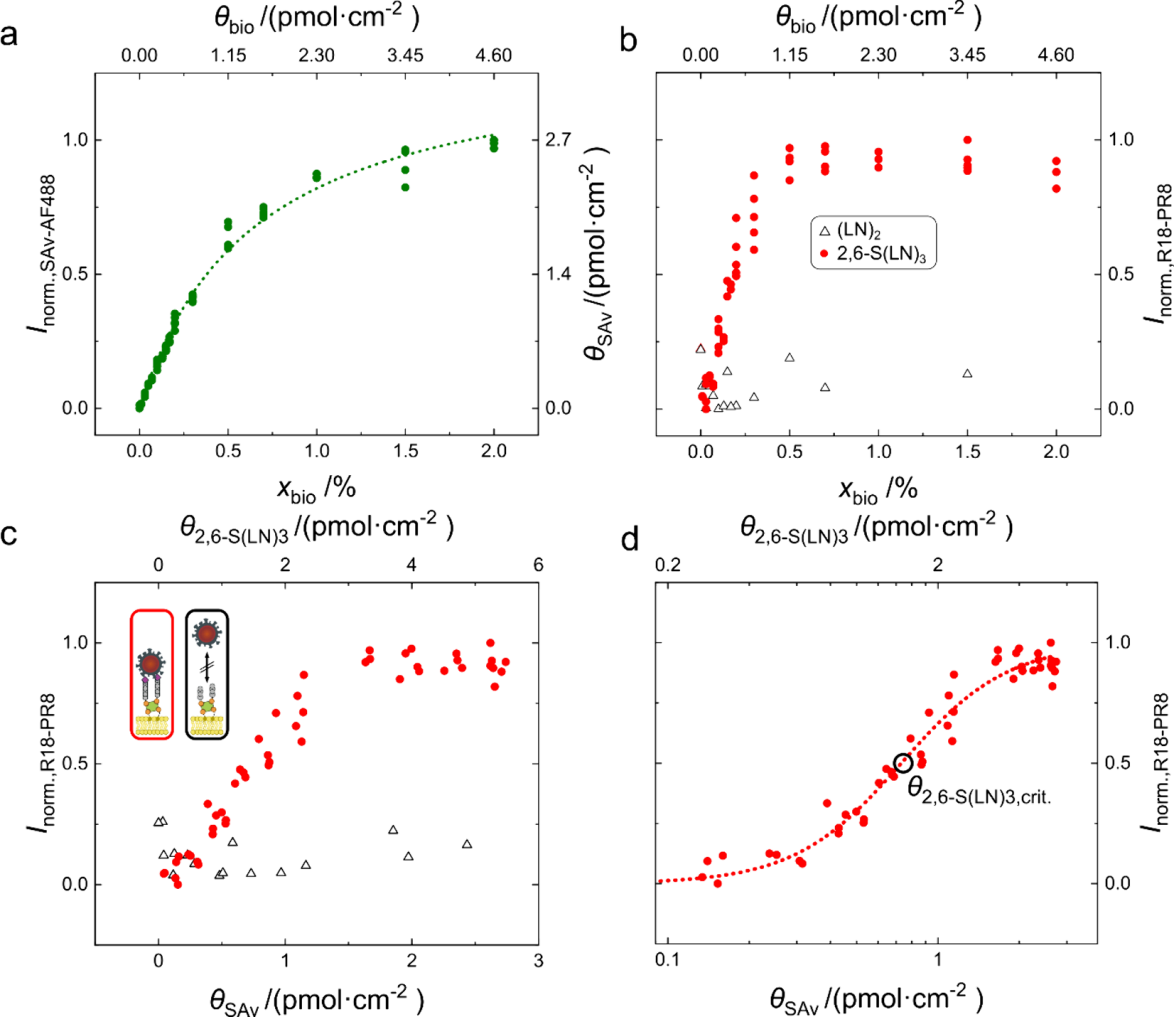
Study of influenza A virus (PR8) binding to an SLB-based glycan array. The binding of PR8 to SLBs functionalized subsequently with SAv and biotinylated (LN)_2_ and 2,6-S­(LN)_3_ was studied. Fluorescence intensities are shown as pixel averages. (a) Normalized, green fluorescence intensity (*I*_norm.,SAv‑AF488_) from SAv-AF488 plotted as a function of *x*_bio_ and θ_bio_. SAv densities θ_SAv_ determined according to the previously described method are given as a right ordinate. The corresponding Langmuir fit is depicted as a dotted line. (b) Normalized, red fluorescence intensity (*I*_norm.,R18‑PR8_) quantifying the amount of rhodamine-labeled virus particles bound to the surface plotted as a function of *x*_bio_ and θ_bio_. (c) *I*_norm.,R18‑PR8_ as a function of θ_SAv_. (d) Data fitted using the Hill equation (eq 1), which allows for determination of the threshold receptor density θ_2,6‑S(LN)_3_,crit_.

As is evident from Figure 5a, good control of the SAv density was achieved, whereby focus was placed on the low range of densities, where the threshold receptor density of the virus binding is to be expected. Accordingly, Figure 5b shows significant binding of the rhodamine-labeled virus particles to the surfaces functionalized with 2,6-S­(LN)_3_, whereas little binding was seen for surfaces functionalized with the control glycan (LN)_2_. This is in agreement with the selectivity of PR8 binding reported in the literature.^9,10,12^ A background signal in the red fluorescence is nevertheless observed that is largely independent of the receptor density. Due to the intense rinsing protocol, this background cannot be attributed to the presence of virus particles in the supernatant. Instead, it is more likely caused by some nonspecific adsorption from the virus sample, since the red intensity generally increased over time to a certain extent independent of the lipid composition. A likely explanation could be the presence of unbound dye in the virus sample upon labeling or the nonspecific sedimentation of larger aggregates of virus particles. This interpretation is supported by the observation that the red fluorescence generally increased independent of the receptor density with the exposure time. The background effect was experimentally mitigated by reducing the incubation times from overnight experiments to 4 h.

Nevertheless, the control of receptor density described above allows for the quantitative analysis of the virus binding. Like the previous studies shown in this work, SAv densities (θ_SAv_) were determined from the fluorescence intensities of SAv-AF488. It can furthermore be estimated that the corresponding receptor density is equal to 2θ_SAv_. The resulting density-dependent plots of the red fluorescence intensities arising from the rhodamine label on the virus particles are shown in Figure 5c,d.

As expected, superselective binding is indicated by the sigmoidal curve found for the virus binding (red fluorescence) as a function of SAv and receptor density featuring a threshold receptor density. A fit of these binding curves according to the Hill equation (eq 1)^61,62^ using a simplified apparent affinity constant for the surface receptor density (*K*_θ,app_) at a constant virus concentration and a varied receptor density results in a threshold receptor density of θ_2,6‑S(LN)_3_,crit._ = 1.4 ± 0.04 pmol·cm^–2^, which is in a similar range to values determined based on the multivalent affinity profiling method (Figure 5d).^9^ Differences might arise from the nonspecific fouling observed in this method or the presence of flow in the case of the multivalent affinity profiling method as opposed to this well-plate-based assay.

1 $$I_{\mathrm{red},\mathrm{norm.}}\propto \theta_{\mathrm{PR}8}=\frac{{\left(K_{\theta ,\mathrm{app}}\theta_{2,6\mathrm{‐S}{\left(\mathrm{LN}\right)}_{3}}\right)}^{n}}{1+{\left(K_{\theta ,\mathrm{app}}\theta_{2,6\mathrm{‐S}{\left(\mathrm{LN}\right)}_{3}}\right)}^{n}}$$

It should furthermore be noted that the Hill equation does not account for steric hindrance and kinetic trapping and is here solely used to provide a good estimate of the threshold receptor density.

In conclusion, this study of a more complex, multivalent virus binding example, concerning both the binding as well as the practical complexity of the sample, presents a second showcase of the excellent applicability of the automated SLB platform method to study complex systems in extensive assays with minimal manual labor. Future use of such a protocol can involve the high-throughput use of such arrays with multiple glycans, multiple glycan densities and multiple virus types, to explore changes in binding affinities within virus series that may arise as a result of mutations.

## Conclusions

We established a fully automated well-plate-based workflow for preparing and functionalizing high-quality SLBs that yields robust, antifouling surfaces in a scalable approach. Systematic process optimization yielded homogeneous bilayers verified by fluorescence microscopy and FRAP measurements. By tuning the mole fraction of biotinylated lipid (*x*_bio_), subsequent binding of streptavidin and biotinylated receptor provided precise control over the receptor density, quantified by fluorescence readout via microscopy and plate reader. A quantitative, statistical assessment showed precise control of the receptor density in the low and sub pmol·cm^–2^ range.

Density-resolved measurements enabled quantitative studies of biorecognition processes. In the case of strong, highly selective DNA hybridization, the density of complementary target ssDNA increased linearly with the receptor density for low coverages. This method provides a practical way to map and tune the operating window for hybridization assays, which can be limited due to electrostatic effects.

Extending to a multivalent binding case, the superselective binding of PR8 virus particles to different glycans was studied. The method allowed for a study of the specificity of binding and furthermore gave quantitative information about the virus coverage as a function of glycan density, which could be compared to previous studies.

Together, these results show that automatically generated arrays of SLBs with control over receptor density provide a general, scalable platform to map and tune biorecognition processes, which can be used to guide surface designs. Unlike rehydration-based SLB array methods that rely on drying and subsequent membrane reformation, the vesicle-fusion approach used here maintains the bilayer in a continuously hydrated state, which reduces the risk of membrane defects, while implying shorter shelf lives of several days and more controlled storage conditions. This work is in line with the ongoing advances in lab automation^44,49^ and standardization and the growing availability of commercial liquid-handling and detection systems, which will increase possible throughput and reliability in the coming years. Coupling such a workflow to integrated plate readers further enables high-throughput screening of diverse receptors and targets.

At the same time, this work highlights intrinsic limitations of SLBs, particularly their sensitivity toward air exposure, which complicates handling and long-term stability. Several strategies have emerged to address this challenge. Polymer-SLBs provide enhanced mechanical robustness and can incorporate additional functions.^63^ Another promising concept involves inverse phospholipids like 2-[[2,3-bis­(oleoyloxy)­propyl]­dimethylammonio]­ethyl hydrogen phosphate (DOCP), which forms stable monolayer–bilayer hybrids through coordination with oxide surfaces that can be applied via inkjet printing.^46^ The incorporation of such concepts to the described workflow can further increase its operational range and find application in biosensing.

The functionalization of surfaces using the biotin–SAv coupling is versatile and well established, providing a reliable route to introduce specific biomolecular interactions at interfaces.^24^ It has been shown that a varied linker length between the lipid headgroup and the biotin moiety can affect the binding stoichiometry.^28,64,65^ Nevertheless, the biotin–SAv coupling imposes an upper limit of achievable receptor densities due to the finite packing of SAv and may compromise the antifouling characteristics of lipid-based surfaces.^28^ To reduce the molecular footprint associated with a receptor and simultaneously reduce nonspecific binding, covalent binding methods, which have been applied in polymer-based approaches of receptor density control,^66–68^ could offer a promising alternative without the need for bulky protein linkers or multistep assemblies. Due to its scalability and variability, the method for the preparation of SLBs shown herein could therefore be adapted to such covalent binding approaches in future studies.

## Experimental Section

### Materials

All solutions were prepared using ultrapure water (ρ > 18 MΩ·cm) obtained from a Milli-Q eq 7000 (Sigma-Aldrich). 2-Propanol (≥99.5%), chloroform (99.8%), and phosphate-buffered saline (PBS) tablets were purchased from Sigma-Aldrich (Zwijndrecht, The Netherlands). Alexa Fluor 488 (AF488) and Alexa Fluor 350 (AF350)-labeled streptavidin (SAv) conjugates were obtained from ThermoFisher Scientific (Landsmeer, The Netherlands). Lipids were acquired from Avanti Polar Lipids (Alabaster, AL, USA): 1,2-dioleoyl-*sn*-glycero-3-phosphocholine (DOPC), 1,2-dioleoyl-*sn*-glycero-3-phosphoethanolamine-*N*-(cap biotinyl) (sodium salt) (biotin-DOPE), and Texas Red 1,2-dihexadecanoyl-*sn*-glycero-3-phosphoethanolamine, triethylammonium salt (TR- DHPE).

Biotinylated single-stranded DNA (5′-ACACACACACACACACACACACACAC-TEG-biotin-3′) and 5′-Alexa Fluor 488-labeled complementary ssDNA were obtained from Integrated DNA Technologies (IDT, Coralville, IA, USA). Biotinylated glycans 2,6-S­(LN)_3_ and (LN)_2_ were provided by Elif Uslu and Geert-Jan Boons and synthesized as described previously.^9^ Influenza A/Puerto Rico/8/34 (H1N1, Mt. Sinai strain) virus samples were prepared, inactivated, and purified by GD Animal Health (Deventer, The Netherlands) following a previously described procedure.^12^ 200 μM Zanamivir (GlaxoSmithKline) was added to the virus samples to inhibit neuraminidase. Virus Single Particle Tracking (SPT) were performed on a custom-built setup according to a previously described method.^69,70^ to estimate particle concentrations. A total of 128 frames were analyzed, in which 4423 particles were detected revealing a stock concentration of 110 pM and a broad size distribution around 150 nm in particle diameter.

### Lipid Vesicles

Lipid mixtures of defined stoichiometry (DOPC, biotin-DOPE), where *x*_bio_ is the molar fraction of biotin-DOPE, were prepared from chloroform stock solutions of the individual lipids. TR-DHPE was only included in selected experiments for fluorescence visualization of the SLBs.

The solvent was evaporated under a gentle stream of nitrogen to form a thin lipid film, which was subsequently dried under vacuum for at least 1 h to remove residual solvent. The dried lipid film was then rehydrated in Milli-Q water to obtain dispersions with a total lipid concentration of 1 mg·mL^–1^. The resulting suspensions were extruded through 100 nm polycarbonate membranes (Avanti Polar Lipids, Alabaster, AL, USA) to yield small unilamellar vesicles (SUVs) of defined size and lipid composition. The dispersions were stored at 4 °C and used within a month. Prolonged storage was found to interfere with the proper formation of supported lipid bilayers (SLBs) via vesicle fusion. According to previous studies, vesicle size and colloidal stability of the SUV dispersions, were tested the SUV dispersions were routinely characterized by dynamic light scattering.^59^

### Liquid Handler

Automated protocols were developed on a JANUS G3 automated liquid handling workstation (PerkinElmer, Waltham, MA, USA) equipped with an 8-tip Varispan arm, 250 μL Cavro syringe pumps and a temperature-controlled stage (CPAC Ultraflat, Industrial Heating & Cooling GmbH, Martinsried, Germany). All solutions were transferred from reagent troughs (for Milli-Q and buffers) or Eppendorf tubes (for other reagents) in appropriate holders to 384-well plates (SensoPlate 384-well plates, F-bottom, glass-bottom, black, Greiner Bio-One, Frickenhausen, Germany) positioned on the temperature-controlled stage. Both 50 μL conductive as well as 50 μL nonconductive tips were used (RoboRack, PerkinElmer, Waltham, MA, USA).

The *x*/*y*/*z* positions of the tips relative to the well-plate bottom were thoroughly calibrated. A distance of 1.5 mm between the well bottom and the pipet tip were found to be optimal. Pipetting speeds were set to 5 μL·s^–1^ for volumes below 10 and 10 μL·s^–1^ for larger volumes, as excessive shear can introduce defects in the formed layers. Prior to each experiment, the instrument fluidics were carefully purged of air bubbles by repeated flush/wash cycles—crucial for both consistent dispensing volumes and the integrity of the SLBs. The introduction of air was further minimized by avoiding any blowout volumes, or transport air gaps. All parameter optimization and method development were carried out using WinPREP software (PerkinElmer, Waltham, MA, USA).

The protocol enables the automatic preparation of 24 SLBs in parallel, which was mostly used to handle triplicates of 8 different lipid compositions, each representing a gradient of *x*_bio_ (see the schematic in Figure S5). The following sections provides general descriptions of the individual steps. For clarity, the modular workflow is divided into subsections covering activation and SLB formation, SLB functionalization and gradient creation as well as the respective case studies on DNA hybridization and virus binding. All details of these steps are provided in Tables S3–S5. Process files can be provided by the authors upon request.

### Surface Activation and SLB Formation

The flat glass-bottom surfaces of 384-well plates were activated with 2 M NaOH at 45 °C for 1 h. After thorough rinsing with PBS, 35 μL of PBS and 5 μL of a 1 mg·mL^–1^ dispersion of SUVs in Milli-Q were added to each well. Up to eight different types of vesicles with different lipid compositions (*x*_bio_) were used. Following an incubation period of 30 min, the wells were rinsed 5 times with PBS (pH 7.3, 137 mM NaCl), followed by 5 additional rinses with Milli-Q water to induce osmotic stress and improve bilayer quality. After a further 10 min of incubation to complete vesicle rupture, the wells were rinsed a final 5 times with PBS, to obtain high quality SLBs (Table S3). The density of the biotinylated lipid in the SLB (θ_bio_) was calculated from the molar surface density of lipids (θ_lipid_) based on the molecular footprint of a DOPC lipid according to previous studies^15,28^ using eq 2.

2 $$\theta_{\mathrm{bio}}=x_{\mathrm{bio}}\theta_{\mathrm{lipid}}=x_{\mathrm{bio}}\times 230\;\mathrm{pmol}\cdot {\mathrm{cm}}^{-2}$$

### Functionalization

In the second part of the protocol, SLBs of various compositions (*x*_bio_) obtained as described above were functionalized by incubating with fluorescently labeled SAv conjugates (AF350-SAv and AF488-SAv) for 45 min. The labeling allows for a fluorescent readout of the receptor density. Following the incubation, the wells were thoroughly rinsed with PBS to remove unbound SAv (Table S4).

### Receptor and Target Binding

In the final part of the workflow, the surfaces showing triplicates of a gradient in *x*_bio_ were applied to biorecognition studies, namely DNA hybridization and virus binding to glycosylated surfaces. The gradients of eight distinct lipid compositions—and thus varying SAv densities—were commonly used to evaluate target binding while including both an appropriate receptor as well as appropriate negative controls in the same assay. Pipette tips were exchanged between different receptor solutions to avoid cross-contamination.

For the DNA hybridization assay, biotinylated single-stranded DNA (ssDNA, 5′-ACACACACACACACACACACACACAC-TEG-biotin-3′) or a biotinylated, scrambled DNA sequence (negative control) were immobilized on the streptavidin-functionalized SLBs (AF350-SAv) for 30 min. After rinsing with PBS to remove unbound DNA, the complementary AF488-labeled ssDNA (target) was introduced at a concentration of 250 nM in PBS. Following a 4 h incubation at room temperature, the surfaces were rinsed thoroughly with PBS to remove nonhybridized strands. Fluorescence readout was performed immediately afterward to quantify both receptor and hybridized target densities.

For the virus binding assays, gradients of AF488-SAv on SLBs were functionalized with the biotinylated glycans (receptors) 2,6-S­(LN)_3_ or (LN)_2_ by incubation with 500 nM glycan solutions in PBS for 30 min. After thorough rinsing with PBS, the surfaces were exposed to a 1.4 pM dispersion of R18-labeled PR8 virus particles (target) in PBS and incubated for 4 h. To inhibit neuraminidase from cleaving glycans, 200 μM Zanamivir (GlaxoSmithKline) was added to the virus samples. Subsequent PBS rinsing removed unbound virus particles, and fluorescence measurements were used to quantify both receptor and bound target densities. After thorough rinsing the surface density of both receptor (based on fluorescently labeled SAv) as well as target were determined by fluorescence readout.

### Fluorescence Readout

The fluorescence intensity was measured both by widefield epifluorescence microscopy and a multimode plate reader. Microscopy was used for method development because it enables direct assessment of bilayer surface quality.

Fluorescence micrographs were acquired on an Olympus inverted IX71 epifluorescence microscope with X-Cite 120PC mercury arc lamp and a digital Olypus DR70 camera. Appropriate filter sets were used for each label: AF350 (330 nm ≤ λ_ex_ ≤ 385 nm; λ_em_ > 420 nm), AF488 (460 nm ≤ λ_ex_ ≤ 490 nm; 505 ≤ λ_em_ ≤ 545) and R18 (510 nm ≤ λ_ex_ ≤ 550 nm; λ_em_ > 590 nm). For illustration, 8-bit RGB images were saved, whereas quantification was performed based on 16-bit greyscale images. Mean fluorescence intensities were computed from multiple regions of interest (ROIs) per well, averaged across wells and independent experiments for each lipid composition. Errors were calculated by propagation of the corresponding standard deviations.

Alternatively, the more scalable, high-throughput method of a plate reader was used to measure fluorescence intensities of labeled SAv conjugates and labeled targets, respectively. Measurements were performed on a TECAN Infinite M Nano+ (Tecan Trading AG, Männedorf, Switzerland) in bottom-read mode using the same spectral window as the microscope. Monochromator settings were AF350 (λ_ex_ = 350 nm ± 10 nm; λ_em_ = 460 nm ± 20 nm), AF488 (λ_ex_ = 485 nm ± 10 nm; λ_em_ = 535 nm ± 15 nm), and R18 (λ_ex_ = 540 nm ± 10 nm; λ_em_ = 600 nm ± 20 nm). All measurements were performed at room temperature.

For the quantification of molar surface densities from data from plate reader or microscopy measurements, normalized average pixel intensities were used. Normalization was done by min–max scaling using the average background intensity (*x*_bio_ = 0%) and maximum averaged fluorescence intensity (*I*_max_). The SAv binding curves (*I*_AF488,norm._ and *I*_AF350,norm._ as a function of *x*_bio_ in Figure 3 b and c) were fitted to Langmuir isotherms (eq 3) yielding maximum normalized intensities (*I*_norm.,max_) corresponding to a saturated SAv layer.

3 $$I_{\mathrm{norm.}}=I_{\mathrm{norm.},\max}\frac{K_{\mathrm{SAv}}x_{\mathrm{bio}}}{1+K_{\mathrm{SAv}}x_{\mathrm{SAv}}}$$

Using the molar surface density of such saturated layer taken from the literature (3.7 pmol·cm^–2^),^28^ SAv densities were calculated according to eq 4.

4 $$\theta_{\mathrm{SAv}}=\theta_{\mathrm{SAv},\max}\frac{I_{\mathrm{norm.}}}{I_{\mathrm{norm.},\max}}=3.7\;\mathrm{pmol}\cdot {\mathrm{cm}}^{-2}\times \frac{I_{\mathrm{norm.}}}{I_{\mathrm{norm.},\max}}$$

### FRAP Measurements

FRAP measurements were carried out using a Nikon A1 confocal microscope (Nikon) with a 20× objective (NA 0.75). Circular ROIs with a diameter of 5 μm were bleached for ∼1.6 s using the 488 and 561 nm LASERs simultaneously at maximum power. Reference ROIs, used for fading correction, were the same size as the bleach ROIs and situated as far as possible from their respective bleach ROI within the imaged region (317 μm × 40 μm). After bleaching, the bleach and reference ROIs were imaged for up to 5 min to record fluorescence recovery. Recovery curves are plotted as ratios of background corrected intensities of bleached ROI/reference ROI as fading correction in time.

To correct for the effect of diffusion occurring together with bleaching, an effective radius, *r*_e_, of the bleach spot was determined from the first image after bleaching and used to correct the diffusion coefficient *D* according to eq 5,

5 $$D=\frac{{r_{n}}^{2}+{r_{e}}^{2}}{8\tau_{1/2}}$$

where *r*_n_ is the nominal radius defining the (circular) bleach spot and τ_1/2_ is the half-recovery time.^53^ Time traces are first background corrected, then fading corrected and subsequently normalized by setting the average intensity before bleaching to 1, after which τ_1/2_ is determined. The effective radius is determined from the (Gaussian) fit of (averaged) line profiles across the center of the initial bleach spot, where *r*_e_ is the radius at 86% of the maximum bleach depth.^53^

## Supplementary Material



## Notes

Detailed descriptions of functional surfaces and lipid compositions, QCM-D experiments verifying layer assembly and binding kinetics, fluorescence-based control of receptor densities and LOD, micrographs for the virus binding assays on glycan-functionalized SLBs, image analysis of the particle tracking method, and detailed protocols for the automated liquid-handling protocols for SLB formation, functionalization, and target binding, including all supplementary figures and tables (PDF)
