# Supplementary material for: Automated Generation of Supported Lipid Bilayer Arrays with Controlled Receptor Densities in Well Plates
Source: ACS Appl Mater Interfaces. 2026 Mar 30;18(13):19946–57. doi: 10.1021/acsami.6c02551 (PMC13067233; doi:10.1021/acsami.6c02551)
Supplement: Supplementary file 1 [file am6c02551_si_001.pdf]

# Supporting Information

# Automated Generation of Supported Lipid Bilayer Arrays with Controlled Receptor Densities in Well Plates

*Jannis Schlicke,*[a] *Jacopo Movilli,*[a] *Dorothee Wasserberg,*[a] *Raphael N. Biendara,*[a] *Samer Aphrham,*[a] *Pascal Jonkheijm,*[a] *Elif Uslu,*[b] *Geert-Jan Boons,*[b] *Robert Molenaar,*[c] *Rick Elbert,*[d] *Sjaak de Wit,*[d] *Erhard van der Vries,*[d] *Jurriaan Huskens**,[a]

[a] Department of Molecules and Materials, MESA+ Institute & TechMed Centre, Faculty of Science and Technology, University of Twente, P.O. Box 217, Enschede 7500 AE, The Netherlands

[b] Department of Chemical Biology & Drug Discovery, Utrecht Institute for Pharmaceutical Sciences, Utrecht University, 3584 CG Utrecht, The Netherlands

[c] NanoBioPhysics Group, MESA+ Institute, University of Twente, 7500 AE Enschede, The Netherlands

[d] Royal GD, Arnsbergstraat 7, Deventer 7418 EZ, The Netherlands

* Email: j.huskens@utwente.nl

**Table S1.** Qualitative comparison of different platforms for the fabrication of SLB assays concerning their scalability, standardization and automation. Scalability refers to the ability to perform large numbers of parallel experiments. Standardization reflects the compatibility with international standards for laboratory equipment. Automation describes the feasibility of integrating the workflow in robotic laboratories.

| **platform** | **manual well-plate SLB assays** | **printed/spotted SLB arrays** | **Microfluidic SLB systems** | **This work: Automated Well-Plate SLBs** |
|---|---|---|---|---|
| scalability | low (manual preparation) | high (multiple spots per substrate) | moderate (limited number of microchannels per chip) | high (scales with the liquid handling platform) |
| standardization | high (standard equipment) | moderate (custom printing platforms and substrates) | low (custom chips) | high (standardized infrastructure) |
| automation | low (manual pipetting) | moderate (robotic spotting systems, readout with microscopy) | moderate (highly automated but complex) | high (fully compatible with established liquid handling) |
| key advantage | widely available infrastructure and high adaptability | automated generation of multiplexed SLB patches | Precise control of flow conditions an gradients | Scalable, adaptable and reproducible SLB preparation using a straight-forward approach and standard lab automation |
| representative references (in main text) | 43 | 46-48 | 36-42 | this work |

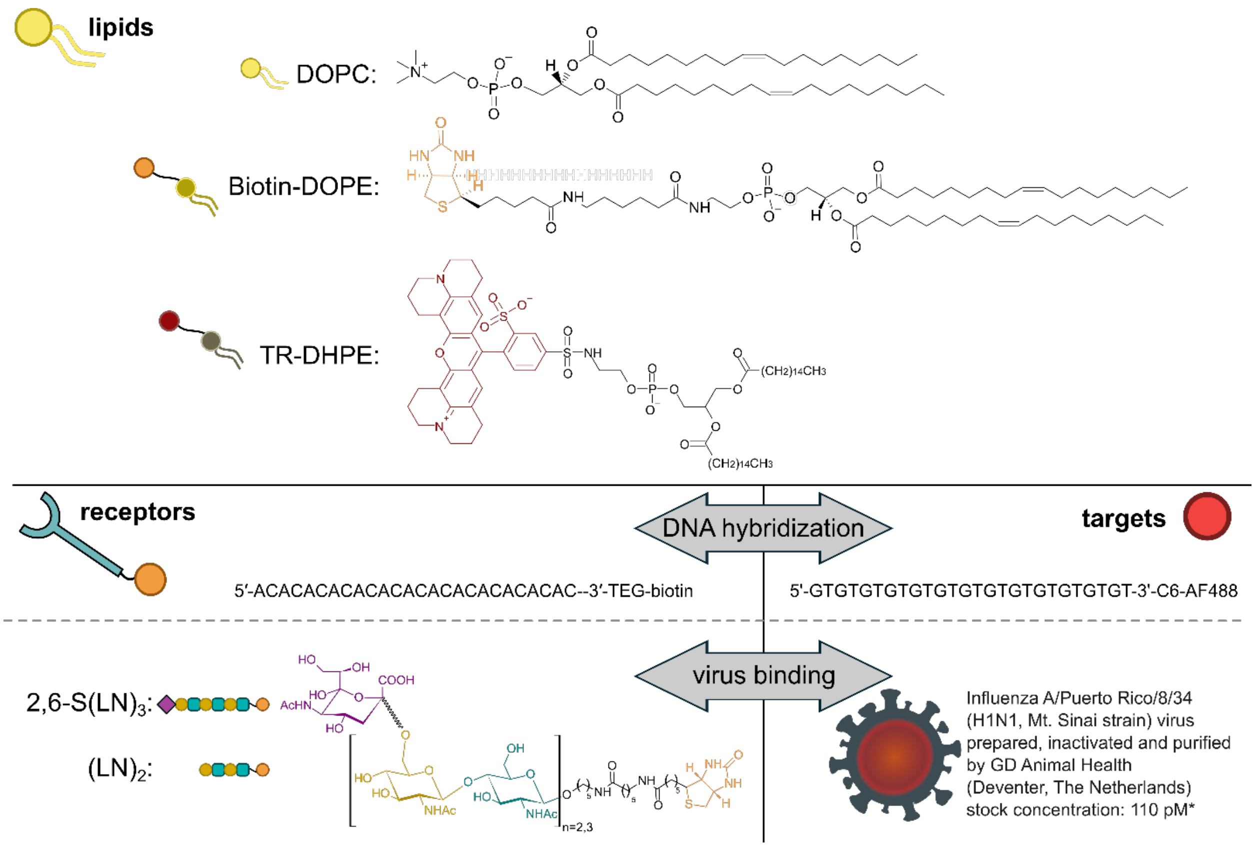


**Figure S1.** Structures and detailed information about the functional surfaces used throughout this work. The involved lipids, receptors and targets are shown. *Stock concentration determined by single particle tracking.

## QCM-D Experiments on the Kinetics of the Assay

Dissipative quartz-crystal microbalance (QCM-D) measurements were performed to monitor and verify the layer assemblies. An example of such experiment is shown in Fig. S2. A QSense4 with 5 MHz Si/$SiO_2$ sensors in flow chambers was used. The respective reactant solutions (see Tables S3-S5 below) were flushed through the flow chambers at 30 μL·min$^{-1}$, and the sensor response was recorded. Despite of significant differences between measurements performed in flow and the conditions in well plates, these experiments verified the layer formation as well as the receptor binding and could furthermore be used to assess incubation times.

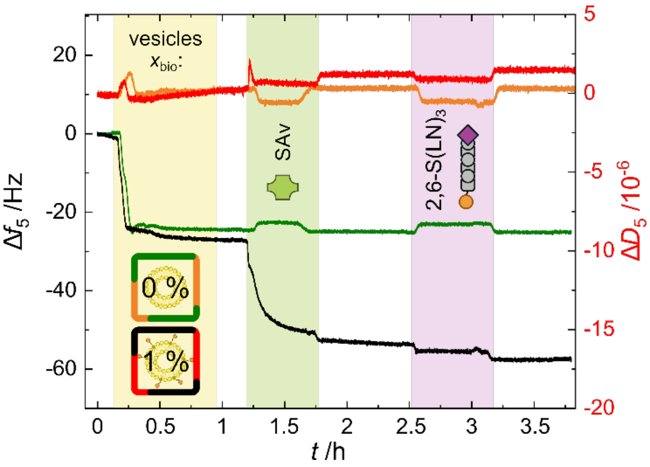


**Figure S2.** QCM-D monitoring of the layer assembly in flow. Changes in frequency and dissipation of the 5$^{th}$ overtone are plotted as a function of time. Different solutions were flushed over 5 MHz Si/$SiO_2$ surfaces (30 μL·min$^{-1}$) to mirror the well-plate protocol (Tables S3-S5).

After the injection of SUV dispersions of different composition ($x_{bio}$ = 0 %, $x_{bio}$ = 1 %), the characteristic response associated with SLB formation was observed, including a frequency shift of approximately 23 Hz (fundamental), consistent with literature for this type of layers.[1, 2] As expected, the subsequent binding of SAv was rapid and specific for $x_{bio}$ = 1 %, whereas only a

small bulk shift and no specific binding were observed for $x_{\text{bio}}$ = 0 %. The frequency change associated with SAv adsorption was in line with near-full coverage of the SLB surface by the protein. Likewise, binding of the biotinylated glycan 2,6-S(LN)$_3$ occurred only for the SAv-coated surfaces ($x_{\text{bio}}$ = 1 %), confirming the surface-chemistry scheme and highlighting the fast equilibration of the SAv/biotin interaction.

**Control of Receptor Densities**

In order to determine the lower limit of detection of the control of SAv densities by the means of lipid composition ($x_{\text{bio}}$) in combination with a fluorescence readout, intentionally low fractions of the biotinylated lipid were investigated in separate experiments. The fluorescence intensities measured for these low concentrations ($x_{\text{bio}} < 0.2$ %) at different exposure times are summarized in Fig. S3 and plotted as a function of both $x_{\text{bio}}$ as well as the receptor density $\theta_{\text{bio}}$, which was calculated by Equation 1. An approximately linear dependence of the intensity on $x_{\text{bio}}$ can be seen for both AF350-SAv as well as AF488-SAv. It is obvious, that both slope as well as signal to noise ratio are significantly higher for AF488-SAv as compared to AF350-SAv. This can be attributed to the higher quantum yield of AF488 as well as lower background fluorescence for the green dye.

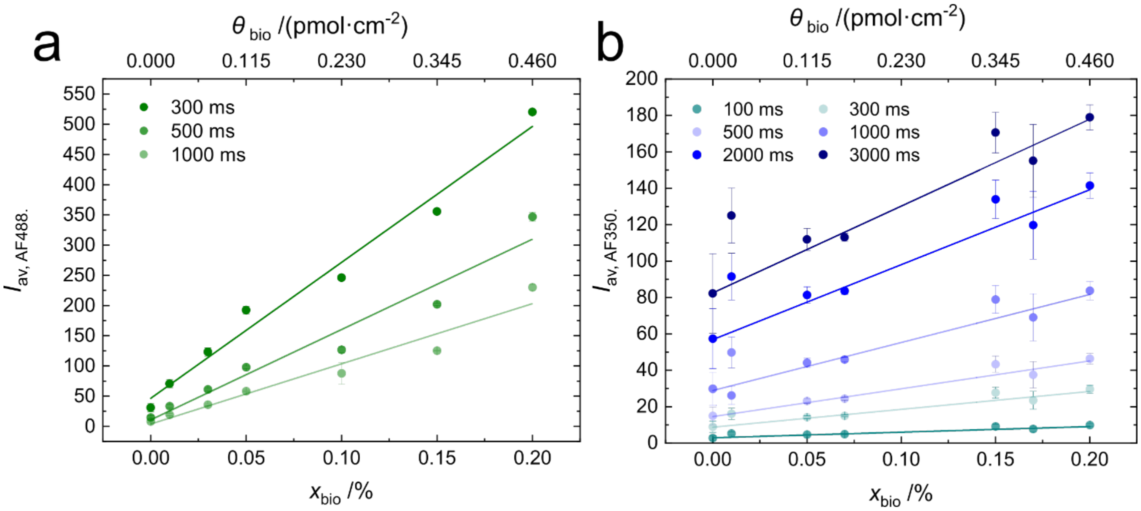


**Figure S3.** Control of receptor density on DOPC/biotin-DOPE SLBs DOPC/biotin-DOPE SLBs with different $x_{bio}$ after the binding of (a, green) SAv-AF488 or (b, blue) SAv-AF350 for $x_{bio} <$ 0.2 %. The amount of bound SAv was visualized by fluorescence microscopy. Normalized mean fluorescence intensities are shown as a function of $x_{bio}$ and the receptor density. Linear regressions were used to determine limits of detection.

The limit of detection was calculated from the linear regressions for $x_{bio} < 0.2$ % according to literature[3]. The limits of detection for the different exposure times are summarized in Table S2. An average of those limits of detection and the corresponding standard deviation are given in the main text.

**Table S2.** Summarized limits of detection for different fluorescent dyes and exposure times.

| **Dye** | **Exposure time** t/ms | **Limit of detection** $x_{bio}$/% | **Limit of detection (pmol·cm$^{-2}$)** $\theta_{bio}$/(pmol·cm$^{-2}$) |
|---|---|---|---|
| AF350-SAv | 100 | 0.094 | 0.22 |
| AF350-SAv | 300 | 0.085 | 0.19 |
| AF350-SAv | 500 | 0.082 | 0.19 |
| AF350-SAv | 1000 | 0.084 | 0.19 |
| AF350-SAv | 2000 | 0.082 | 0.19 |
| AF350-SAv | 3000 | 0.082 | 0.19 |
| AF488-SAv | 300 | 0.014 | 0.032 |
| AF488-SAv | 500 | 0.0080 | 0.018 |
| AF488-SAv | 1000 | 0.0081 | 0.019 |

**Virus samples bound to glycan array**

Figure S4 shows exemplary results for the binding of R18-labelled PR8 virus samples to the SAv gradients functionalized with different glycans. Specific binding to the surfaces functionalized with the receptor 2,6-S$(LN)_3$ is evident. Some non-specific binding to the negative control can be seen, which is independent of the lipid composition.

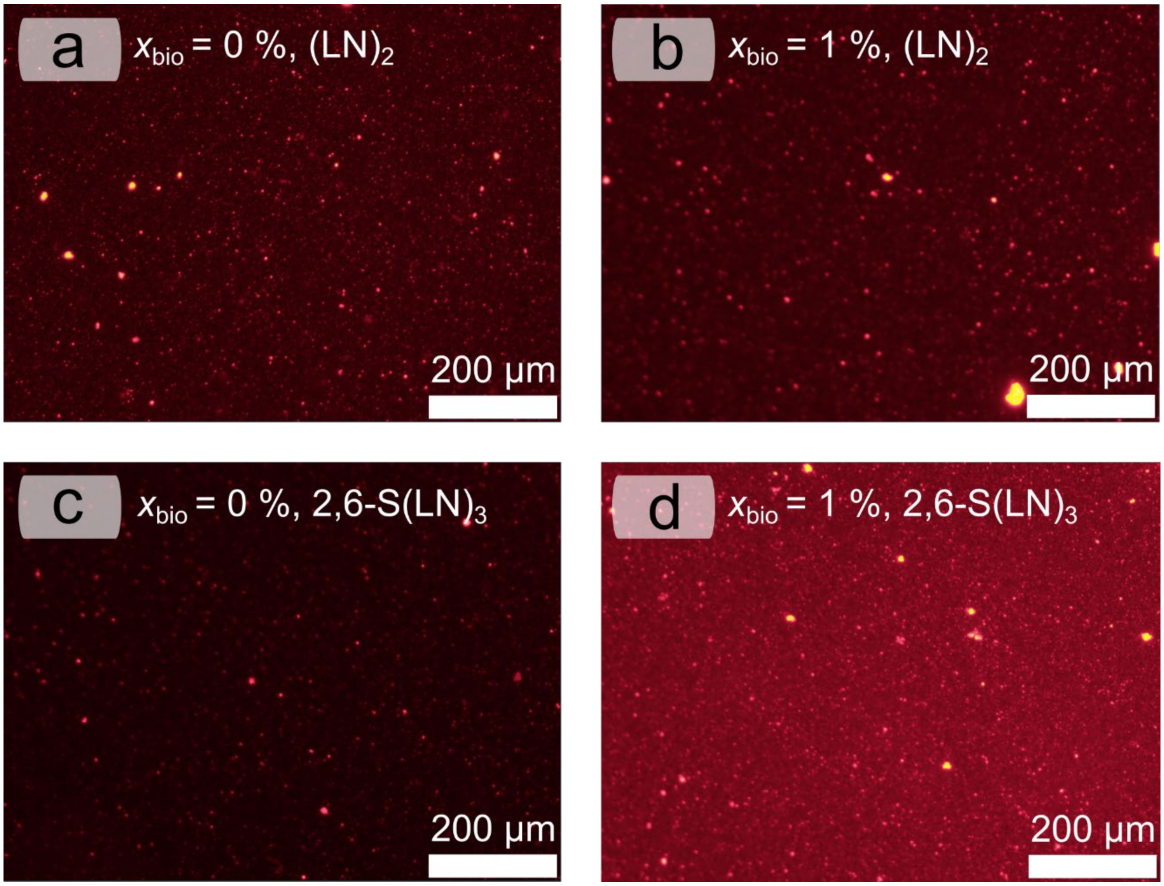


**Figure S4.** Virus (PR8) binding assay on glycan-functionalized SLBs. Lipid composition: $x_{bio}$ = 0 % (a, c) or $x_{bio}$ = 2% (b, d). Receptors: $LN_2$ (a, b) or 2,6-S$(LN)_3$ (c and d). Fluorescence micrographs were acquired after incubation with a 6 pM PR 8 (R18-labelled) sample in PBS with 200 µM Zanamivir, followed by thorough rinsing.

Fluorescently labeled virus samples were pipetted into a 70 μL chamber that has a #1.5H coverslip bottom. This sample chamber mounted on an inverted microscope (Nikon TE-2000U) equipped with a custom in-coupling for a multimode 520 nm, 1.2 W laser diode (Laserland, A-G1000F-C). Excitation light was directed through the microscope backport and focused onto the objective's back focal plane (Nikon, CFI PlanApo 60x NA1.2 Wi). The microscope filter cube contained an excitation filter to remove any wavelength above 520nm from the laser source (Semrock, FF01-513/13-25). A dichroic mirror (Semrock FF538-FDi01) was used to spectrally separate the excitation from the emitted light. The emission was additionally filtered by a long-pass filter that blocks light below 561 nm (Semrock, BLP02-561R-25). Virus fluorescence was registered by a 12-bit camera (Basler, acA2440-75um) using 2x2 pixel binning. The excitation was synchronized to a 1 ms or 2 ms exposure time at framerates of 75 frames$\cdot s^{-1}$ . The illuminated volume was approximately (140 × 60 × 3) $\mu m^3$ with a maximum power density of 1.25 kW $cm^{-2}$. Videos were recorded with a custom-written LabView program.

Several tools are available to locate, count and track objects in videos. These tools are mostly based on the widely-used Crocker–Grier[4] algorithm. Here we used *Trackpy*[5] a well-documented Python implementation of the Crocker–Grier particle tracking algorithm, to identify and track fluorescently labelled virus particles over time. To exclude false positives arising from e.g. noise or (far) out of focus signal, an intensity threshold was applied and the identified viruses were required to be present for a least 5 consecutive frames for tracking.

## Automated Liquid Handling Protocol to Make SLBs

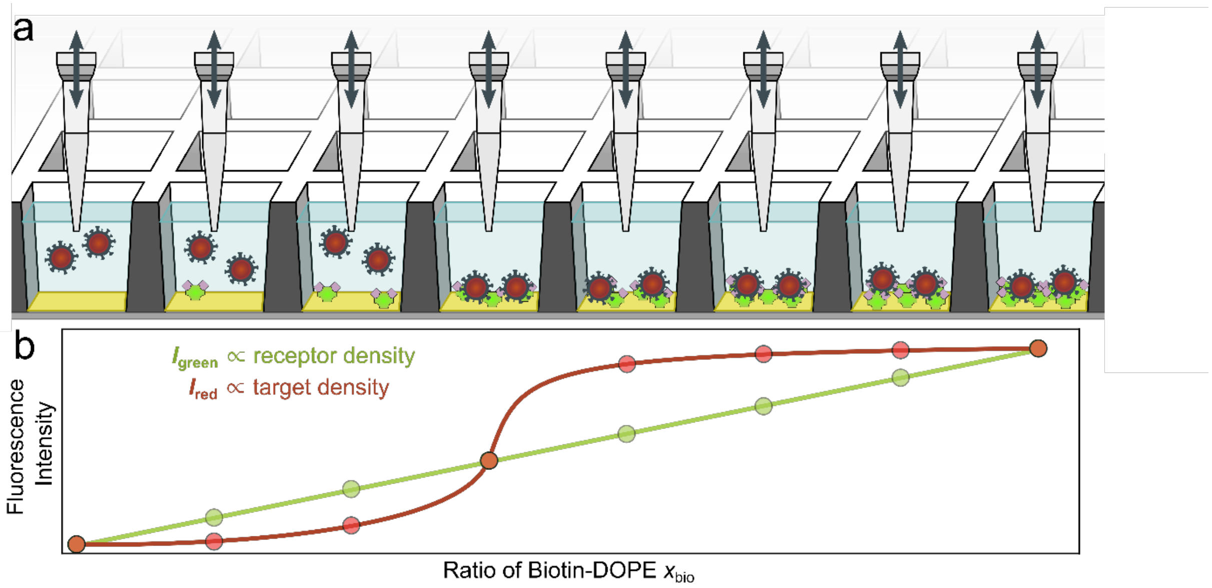


**Figure S5.** The ratio of biotinylated lipid is used effectively to tune the receptor density in separate wells. **a** Triplicates of these density gradients were prepared. **b** Fluorescence labelling of SAv and the target allows for a quantitative study of binding. In case of virus particles, the target density should show a sigmoidal curve indicating a superselective binding.

**Table S3.** Automated protocol for surface activation and supported lipid bilayer (SLB) formation in 384-well plates using a liquid handling workstation. The table lists each procedure, corresponding reagent, dispensed or withdrawn volume, and step type. Repeated steps are indicated as (×*N*), where *N* denotes the number of repetitions. *Procedures marked with an asterisk were performed outside the well.*

| **Procedure** | **Reagent** | **Volume /µL** | **steps** |
|---|---|---|---|
| flush/wash<br>get tips<br>T-controller to 45 °C | | | |
| dispense NaOH | 2M NaOH | +30 | aspire/dispense |
| rinse tips | (Milli-Q to waste)* | (±45)* | aspire/dispense |
| wait 1 h<br>T-controller to 22 °C | | | |
| remove NaOH | liquid in well | -25 | aspire/dispense |
| rinse wells (x10) | PBS<br>liquid in well<br>liquid in well | +40<br>±25<br>-40 | aspire/dispense<br>mix (x7)<br>aspire/dispense |
| drop tips<br>get tips | | | |
| dose buffer | PBS | +35 | aspire/dispense |
| rinse tips | (PBS to waste)* | (±45)* | aspire/dispense |
| add vesicles | LUVs (1 mg·mL$^{-1}$ in Milli-Q) | +5 | aspire/dispense |
| mix vesicles | Liquid in well | ±25 | mixing (7x) |
| wait 30 min | | | |
| rinse wells (x5) | PBS<br>liquid in well<br>liquid in well | +40<br>±25<br>-40 | aspire/dispense<br>mix (x5)<br>aspire/dispense |
| rinse wells (5x) | MQ<br>liquid in well<br>liquid in well | +40<br>±25<br>-40 | aspire/dispense<br>mix (x5)<br>aspire/dispense |
| wait 10 min | | | |
| rinse wells | PBS<br>liquid in well<br>liquid in well | +40<br>±25<br>-40 | aspire/dispense<br>mix (x5)<br>aspire/dispense |
| drop tips | | | |

**Table S4.** Automated protocol for the functionalization of SLBs as a continuation of the procedure shown in Table S3. The table lists each procedure, corresponding reagent, dispensed or withdrawn volume, and step type. Repeated steps are indicated as (×*N*), where *N* denotes the number of repetitions. *Procedures marked with an asterisk were performed outside the well.*

| **Procedure** | **Reagent** | **Volume /µL** | **steps** |
|---|---|---|---|
| get tips | | | |
| dispense SAv | 0.17 µM in PBS | +15 | aspire/dispense |
| | liquid in well | ±25 | mix (x5) |
| wait 45 min | | | |
| remove excess | liquid in well | -15 | aspire/dispense |
| | PBS | +40 | aspire/dispense |
| rinse wells (x25) | liquid in well | ±25 | mix (x7) |
| | liquid in well | -40 | aspire/dispense |
| drop tips | | | |

**Table S5.** Automated protocol for the functionalization of supported lipid bilayers (SLBs) with biotinylated receptors and subsequent target binding, as a continuation of the procedure shown in Table S4. In this part of the workflow, different receptors were often immobilized in separate groups of eight wells, each representing a gradient of $x_{\mathrm{bio}}$, rather than performing triplicate measurements. This allowed for the inclusion of appropriate controls within the same assay. The table lists each procedure, corresponding reagent, dispensed or withdrawn volume, and step type. Repeated steps are indicated as (×*N*), where *N* denotes the number of repetitions. *Procedures marked with an asterisk were performed outside the well.*

| **Procedure** | **Reagent** | **Volume /µL** | **steps** |
|---|---|---|---|
| get tips | | | |
| Add receptor | 1 µM biotin-ssDNA in PBS<br>or 4 µM biotin-glycan in PBS | +5 | aspire/dispense |
| mixing | liquid in well | ±25 | mix (x5) |
| Incubate for 45 min | | | |
| rinse wells (x10) | PBS<br>liquid in well<br>liquid in well | +40<br>±25<br>-40 | aspire/dispense<br>mix (x7)<br>aspire/dispense |
| drop tips<br>get tips | | | |
| Add target | 1 µM AF488-c-ssDNA in PBS or 11 pM PR8 virus (R18) in PBS | +35 | aspire/dispense |
| mixing | Liquid in well | ±25 | mixing (5x) |
| Wait for 4 h | | | |
| rinse wells (x25) | PBS<br>liquid in well<br>liquid in well | +40<br>±25<br>-40 | aspire/dispense<br>mix (x5)<br>aspire/dispense |
| drop tips | | | |

**References**


(1) Di Iorio, D.; Lu, Y.; Meulman, J.; Huskens, J. Recruitment of receptors at supported lipid bilayers promoted by the multivalent binding of ligand-modified unilamellar vesicles. *Chem Sci* **2020**, *11*, 3307-3315.

(2) Di Iorio, D.; Verheijden, M. L.; van der Vries, E.; Jonkheijm, P.; Huskens, J. Weak multivalent binding of influenza hemagglutinin nanoparticles at a sialoglycan-functionalized supported lipid bilayer. *ACS Nano* **2019**, *13*, 3413-3423.

(3) Armbruster, D. A.; Pry, T. Limit of blank, limit of detection and limit of quantitation. *Clin Biochem Rev* **2008**, *29*, S49.

(4) Crocker, J. C.; Grier, D. G. Methods of digital video microscopy for colloidal studies. *J Colloid Interface Sci* **1996**, *179*, 298-310.

(5) *soft-matter/trackpy: v0.7*; Zenodo: 2025. https://doi.org/10.5281/zenodo.16089574 (accessed.02.03.2026)
